# Associations between systemic inflammation, neighborhood tree canopy, and white matter hyperintensities: The Healthy Brain Initiative

**DOI:** 10.1002/alz70860_104126

**Published:** 2025-12-23

**Authors:** Lauren G Toledo, Lilah M Besser, Elaine Le, Mahesh S Joshi, James E. Galvin

**Affiliations:** ^1^ University of Miami Miller School of Medicine, Boca Raton, FL, USA; ^2^ University of Miami, Boca Raton, FL, USA

## Abstract

**Background:**

Systemic inflammation has been associated with greater white matter hyperintensities (WMH), increasing risk for cerebrovascular disease and dementia. Neighborhood greenspaces (e.g., parks) have been associated with reduced inflammation and dementia risk and fewer WMH. Our study addresses a gap in the literature by investigating whether systemic inflammation and neighborhood tree canopy are independently associated with and interact to influence WMH.

**Method:**

We used cross‐sectional data on 161 participants in University of Miami's Healthy Brain Initiative, restricting to ≥ 50‐year‐olds with no to mild cognitive impairment. U.S. Forest Service tree canopy data (2021) were used to derive tree canopy percentage (i.e., area of leaves, needles, branches, and stems covering ground) per Census tract. High‐sensitivity C‐reactive protein (hs‐CRP), a blood‐based biomarker for systemic inflammation, was categorized as low/moderate (0‐3 mg/L) and high (>3 mg/L). Using structural MRI, total WMH volume was divided by total intracranial volume to produce normalized measures of WMH % that accounted for head size differences. Multivariable linear regression models, accounting for clustering by Census tract, tested independent associations between CRP and WMH and between tree canopy and WMH. A similar regression analysis was conducted to investigate the association between a four‐category CRP‐tree canopy variable and WMH (based on median cut points: higher CRP‐lower tree canopy, higher CRP‐higher tree canopy, lower CRP‐lower tree canopy, lower CRP‐higher tree canopy). Models controlled for demographics (e.g., age, sex, race/ethnicity), cognitive status, population density, and area deprivation index.

**Result:**

Participants were 68.7±9.3 years old; 70% were female; 8% identified as Black, 89% White, as 3% other racial group; and 14% identified as Hispanic. Higher CRP was associated with greater WMH (estimate=0.62, 95% CI=0.12, 1.12). Greater neighborhood % tree canopy was associated with fewer WMH (estimate=–0.12, 95% CI=‐0.17, ‐0.08). Compared to those with higher CRP and lower % tree canopy, those with higher CRP and higher % tree canopy had fewer WMH (estimate=‐1.16, 95% CI=‐2.12, ‐0.20).

**Conclusion:**

High systemic inflammation and high neighborhood tree canopy were independently associated with greater and lower WMH, respectively. In those with higher systemic inflammation, having greater neighborhood tree canopy was associated with lower WMH volume.

